# Assembly Mechanisms and Functional Adaptations of Soil Fungal Communities of Different Plant Rhizospheres in Ilmenite Mining Area

**DOI:** 10.3390/jof11030165

**Published:** 2025-02-20

**Authors:** Sumin Chen, Bingliang Liu, Yunfeng Zhang, Lianxin Peng, Liang Zou, Jie Cheng, Qiang Li

**Affiliations:** Key Laboratory of Coarse Cereal Processing, Ministry of Agriculture and Rural Affairs, Sichuan Engineering & Technology Research Center of Coarse Cereal Industrialization, School of Food and Biological Engineering, Chengdu University, Chengdu 610106, China; chensumin76@163.com (S.C.); liubingliang@cdu.edu.cn (B.L.); zhangyunfeng@cdu.edu.cn (Y.Z.); penglianxin@cdu.edu.cn (L.P.); zouliang@cdu.edu.cn (L.Z.)

**Keywords:** plants, ilmenite, rhizosphere soil, fungal community diversity

## Abstract

This study investigated the mechanisms of structural assembly and functional adaptations of fungal communities in the rhizosphere soils of seven different plants grown in the ilmenite zone. We analyzed changes in the rhizosphere soil fungal communities using ITS sequencing. The results revealed that different plants affected the properties of the rhizosphere soil. The contents of organic matter, total nitrogen, and total potassium in the rhizosphere soil exhibited significant variations compared to the soil that was not occupied by plants. Soil fungal composition analysis revealed that *Ascomycota* and *Basidiomycota* were the dominant phyla in the soil of this mining area. At the genus level, compared to the mineral soil without plants, the proportion of *Epicoccum* increased in the rhizosphere soils of different plants, while the proportion of *Fusarium* decreased. Alpha diversity studies revealed that fungal diversity in the rhizospheres of different plants changed significantly. Beta diversity studies showed a significant differentiation in the fungal community structure of different plant rhizosphere soils compared to the KB group. The FunGuild predictions indicated that different plant rhizosphere soils are enriched with different guilds and trophic patterns of fungi. In addition, we found that soil physical and chemical properties were significantly correlated with the abundance and diversity of fungal communities. The above results indicate that plant species and soil physicochemical properties are important factors influencing the assembly of soil fungal communities in the rhizosphere. This research provides insights into the assembly mechanisms and functional adaptations of fungal community structures in the rhizosphere soils of seven plant species in ilmenite iron mining areas. This helps us to screen plant and fungal community assemblages that can promote soil restoration in ilmenite mining areas and provide a theoretical basis for future ecological restoration in ilmenite areas.

## 1. Introduction

With the acceleration of industrialization, the exploitation of mineral resources has caused significant impacts on natural ecosystems, and the ecological and environmental problems in the ilmenite area are particularly prominent. Mining activities in mining areas usually lead to higher concentrations of heavy metals (such as Fe, Ti, Cu, and Pb) in the soil, changes in pH value, decreased organic matter content, and poor soil structure [1,2,3]; these factors have a significant effect on the survival and activity of soil microbial communities [4]. With increasing industrialization, the demand for ilmenite in the chemical industry is increasing [5]. Ilmenite, which is mainly composed of Ti and Fe oxides, is an important mineral that is widely used in the extraction of Ti metal, construction materials, and the chemical industry [6,7,8]. However, the environmental impact of mining activities has attracted much attention [9]. Relevant studies have shown that ilmenite mining can lead to severe pollution of the surrounding soil, water, and atmosphere [10], and the composition and function of soil microbes may change significantly [11,12]. Moreover, the uniquely high heavy metal content and harsh physicochemical conditions in mining areas often lead to a significant decrease in the diversity and functionality of fungal communities [13], thus further exacerbating the degradation of soil ecosystems [14].

The use of phytoremediation to improve soil properties and enhance microbial diversity in mining areas is an effective and sustainable approach to addressing ecological damage caused by mining and other activities in these areas [15,16]. Plant species not only reduce the heavy metal content in the soil but also improve soil nutrients and promote soil material cycling [17]. Some studies have shown that certain plants can promote the activation and migration of heavy metals in the soil by secreting organic substances with chelating effects. These plants also attract and enrich fungal species with heavy metal tolerance [18,19]. In addition, the interaction between plant roots and rhizosphere fungi also provides favorable conditions for the diversity and function of the fungal community [20]. The rhizosphere fungal communities are indispensable components of ecosystems and play key roles in soil nutrient cycling, organic matter decomposition, and plant growth promotion [21,22,23]. Some rhizosphere fungi can secrete substances such as organic acids, metal carriers, and 1-aminocyclopropane-1-carboxylate (ACC) deaminase, which can bind to heavy metals and reduce their toxicity to plants [19,24]. Previous studies have shown that the physicochemical properties of soil are closely related to the microbial communities in the rhizosphere [25,26]. For example, soils with different pH values, organic matter contents, and nutrient components (N, P, etc.) contain different rhizosphere fungal communities [27]. In addition to the physicochemical properties of soil, different plant species are also important factors for the rhizosphere fungal community. Yang et al. studied the diversity of rhizosphere soil microbial communities from four different plants (*Sedum alfredii*, *Rubus hunanensis*, *Lysimachia christinae*, and *Clinopodium gracile*). The results revealed that the rhizosphere microbial composition of *S. alfredii* was significantly different from that of the other three plants [28]. Some studies have shown that plants can also affect the physicochemical properties of the rhizosphere soil, indirectly influencing the diversity of rhizosphere fungi [29].

The rhizosphere is an important site where plants interact with soil microorganisms and is also a key area that affects the functioning of soil ecosystems [30]. Ling et al. found that the rhizosphere of plants is enriched with a large number of microbial communities, and the function of these communities depends on the supply of organic matter from the plant host [31]. Zhang et al. analyzed the inter-root microbial activity of different vegetation types on the Loess Plateau by means of the soil microbial index (RSMI) and found that the re-vegetated soils all showed higher RSMI compared to the control group [32]. There are also studies that show that plants can use some root exudates to directly influence the diversity of microorganisms in the soil [33]. In the ilmenite area, there are differences in the adaptability and tolerance of different plant species to soil environments, which may lead to significant diversity in the structure and function of the soil fungal community in the root zone. Therefore, studying the structure and function of soil fungal communities in the root zones of different plant species in the ilmenite area is of great significance for revealing the interaction mechanisms between plants and microorganisms, evaluating the health status of soil ecosystems, and guiding ecological restoration in mining areas.

In this paper, we used ITS high-throughput sequencing technology to investigate the assembly mechanism of the structure and function of the rhizosphere soil fungal community of seven different plants (*Polygonum plebeium* R. Br., *Tournefortia sibirica* L., *Casuarina equisetifolia* L., *Pteris vittata* L., *Dodonaea viscosa* Jacquem., *Dryopteris coreano-montana* Nakai, and *Alhagi camelorum* Fisch.) in the ilmenite zone. We first determined the physicochemical properties of the rhizosphere soils of different plants and then analyzed the changes in rhizosphere soil fungal community structure among different plants based on α-diversity and β-diversity. Finally, we further analyzed the correlation between soil physicochemical properties and fungal genera. To the best of our knowledge, this is the first time that the effects of different plants on the fungal community in the ilmenite zone have been reported. This provides a reference for a comprehensive understanding of the effects of different plants on the rhizosphere soil microbiomes, and it also serves as an important reference for screening specific plant and fungal community combinations for remediation of soil contaminated by ilmenite mining.

## 2. Methods and Materials

### 2.1. Soil Sample Collection from Seven Plant Species

After preliminary investigations, we found seven widely distributed and well-adapted plants in the ilmenite area (approximately 0.5679 square kilometers) near Wuding County, Chuxiong Yi Autonomous Prefecture, Yunnan Province. These plants include *Polygonum plebeium*, *Tournefortia sibirica*, *Casuarina equisetifolia*, *Pteris vittata*, *Dodonaea viscosa*, *Dryopteris coreano-montana*, and *Alhagi camelorum*. Subsequently, we carefully planted seven plants around the perimeter of the ilmenite mine and selected a 1-square-meter area to plant six plants of each species, with each planting area spaced 8 feet apart. After eight months of careful cultivation, the plants gradually adapted to the special soil conditions of the mine. After ensuring that they were growing well, we took the following steps to collect soil samples: first, we carefully pulled out the roots of the plants; then, we used a sterile brush to accurately collect about 200 g of soil from the roots of each plant and placed them in sterile centrifuge tubes to serve as soil samples. We acquired *Polygonum plebeium*, *Tournefortia sibirica*, *Casuarina equisetifolia*, *Pteris vittata*, *Dodonaea viscosa*, *Dryopteris coreano-montana*, and *Alhagi camelorum* rhizosphere soils named Ppl, Tsi, Ceq, Pvi, Dvi, Dco, and Aca, respectively. In addition, we collected 200 g of soil as a control sample, named KB, from an area near the mine site where no plants were growing. Three biological replicates were set up for each soil sample to ensure the reliability and reproducibility of the experimental results.

### 2.2. Determination of the Soil Samples

The soil pH was measured using a glass electrode method. The available potassium (AK) content of the solutions was measured by flame atomic absorption spectrometry, soil organic carbon (OC) content was determined by the potassium dichromate oxidation–external heating method, total nitrogen (TN) content was assayed through sulfuric acid accelerator digestion and the Kjeldahl method, total phosphorus (TP) content was evaluated using the molybdenum antimony–ascorbic acid spectrophotometric method, total potassium (TK) content was measured with a flame photometer, available nitrogen (AN) content was determined by the alkaline hydrolysis diffusion method, and soil organic phosphorus content was assessed via extraction with ammonium fluoride–hydrochloric acid and sodium bicarbonate solutions, followed by the molybdenum–antimony colorimetric method. Concentrations of metallic elements such as copper, lead, and zinc were determined by inductively coupled plasma–atomic emission spectrometry [34].

### 2.3. DNA Extraction

For microbial diversity analysis, 5 g of each of the rhizosphere soil samples was weighed. Subsequently, 24 soil samples were transported to the laboratory under low-temperature conditions for the purpose of DNA extraction and 16S rRNA sequencing. Extraction of genomic DNA from the soil samples was performed using a dedicated soil DNA extraction kit (D5625-02, manufactured by OMEGA, located in Los Angeles, CA, USA). Following extraction, the DNA was incubated in a 1% (*w*/*v*) agarose gel to ascertain its quality [35].

### 2.4. PCR Amplification and Detection

An aliquot of the extracted DNA was placed in a centrifuge tube and diluted to a concentration of 1 ng/μL with sterile water. This diluted genomic DNA served as the template for amplifying the ITS1 region of the samples, utilizing specialized primers embedded with a barcode (specifically, primer 1737F: 5′-GGAAGTAAAAGTCGTAACAAGG-3′ and primer 2043R: 5′-GCTGCGTTCTTCATCGATGC-3′). The resulting PCR products underwent detection through 2% agarose gel electrophoresis, followed by mixing equal volumes based on their respective concentrations. After thorough mixing, the PCR products were re-examined using 2% agarose gel electrophoresis. Recovery of the products was facilitated by a GLUE recovery kit supplied by the company.

### 2.5. Library Preparation, Sequencing Procedures, and Data Analysis

The library was constructed using the NEBNext^®^ Ultra™ II DNA Library Prep Kit (Illumina, San Diego, CA, USA), and subsequently underwent quantification via Qubit and Q-PCR methods. Qualified libraries were subjected to computer sequencing via the NovaSeq 6000 (Illumina, San Diego, CA, USA) platform. The raw data obtained by sequencing constitute a small proportion of the dirty data. To make the results of the information analysis more accurate and reliable, a data quality control operation was performed: Initially, the raw data for each sample were extracted by segmenting based on the barcode, subsequently eliminating both the barcode and primers. This was then followed by the merging of R1 and R2 sequence data using FLASH software (V 1.2.7, http://ccb.jhu.edu/software/FLASH/, accessed on 1 January 2025) to produce the Raw Tags [36]. After obtaining the qualified Raw Tags, they underwent quality assurance through the use of fastp software (V 0.23.4), resulting in high-quality, cleaned tags. Subsequently, Vsearch software (V 2.24.2) was employed to match these cleaned tags against the database, with the aim of identifying and eliminating chimeric sequences. This resulted in obtaining effective tags that could be used for subsequent analyses [37].

### 2.6. Noise Reduction and Species Annotation

The DADA2 method was used for noise reduction [38]. The quality-controlled effective tags were denoised, and sequences with an abundance of less than 5 were filtered out [39]. The generated sequences were amplicon sequence variants (ASVs) [40]. The ASVs obtained were analyzed using the classify-sklearn algorithm in QIIME 2 to match them with the database and retrieve species information for each ASV [41,42]. Utilizing the ASV annotation results and the sample-specific characteristic table, a table depicting species abundance was compiled, categorizing the data at various taxonomic levels including Kingdom, Phylum, Class, Order, Family, Genus, and Species. Using the species abundance table, we conducted an analysis of the relative abundance of species and carried out clustering based on their abundance.

### 2.7. Alpha and Beta Diversity Analysis

We calculated various alpha diversity indices (such as Observed_otus, Shannon, Simpson, Chao1, Goods coverage, Dominance, and Pielou_e) for different samples using QIIME 2 software. Additionally, we plotted the rarefaction curve and species accumulation boxplot to visualize these data. Differences between samples (or groups) were assessed using beta diversity index analysis, alongside multivariate statistical techniques including Principal Coordinate Analysis (PCOA) and Nonmetric Multidimensional Scaling (NMDS) [43,44].

### 2.8. Functional Prediction and Correlation Analysis

The ecological functions of the corresponding fungi were obtained according to the classification of fungal species using the FunGuild tool (V 1.1) [45]. Prior to conducting the functional cluster analysis, Principal Component Analysis (PCA) was executed, with the original variables being reduced in dimensionality using the FactoMineR (V 2.11) and ggplot2 (V 3.5.1) packages [46]. The statistical software SPSS version 19 was utilized to investigate the correlation between soil physicochemical properties and the ten most abundant fungal genera.

### 2.9. Statistical Analysis

In order to determine the statistical significance of the differences between samples, we conducted a rigorous analysis. Specifically, for comparisons between two sample groups, we used the *t*-test, while for comparisons between more than two sample groups, we used the Tukey test. Differences were considered statistically significant if the *p* value was less than 0.05.

## 3. Results

### 3.1. Soil Physicochemical Measurements

#### 3.1.1. Physical and Chemical Indicators of Rhizosphere Soil

**Figure 1:**
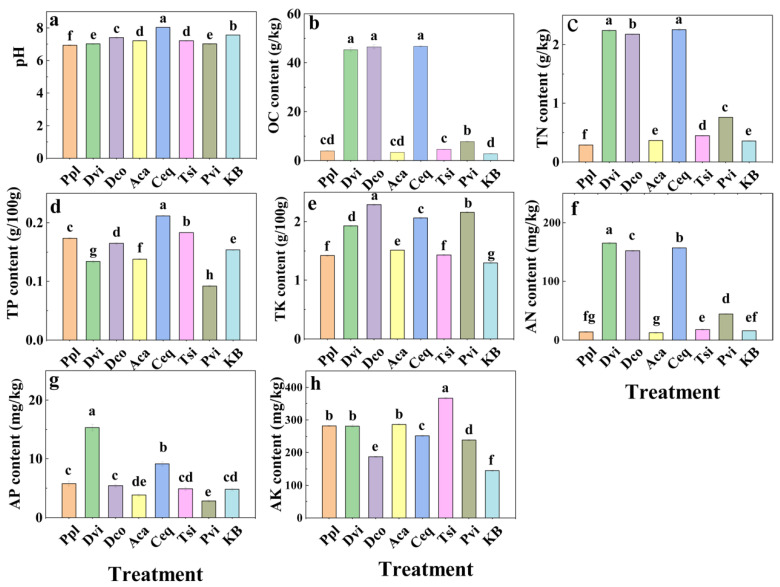
pH (**a**), OC (**b**), TN (**c**), TP (**d**), TK (**e**), AN (**f**), AP (**g**), and AK (**h**) contents of rhizosphere soil samples. Different letters indicate significant differences between samples (*p* < 0.05). KB, soil sample from the ilmenite area without plants; Ppl, rhizosphere soil of *Polygonum plebeium*; Dvi, rhizosphere soil of *Dodonaea viscosa*; Dco, rhizosphere soil of *Dryopteris coreano-montana*; Aca, rhizosphere soil of *Alhagi camelorum*; Ceq, rhizosphere soil of *Casuarina equisetifolia*; Tsi, rhizosphere soil of *Tournefortia sibirica*; and Pvi, rhizosphere soil of *Pteris vittate*.

Among the rhizosphere soils of the seven plant species in the ilmenite area, the pH value of the rhizosphere soil of Ceq was the highest at 8.02, while the pH of the rhizosphere soil of Pvi was the lowest (Figure 1a). The KB group exhibited the lowest soil organic matter content, whereas the Ceq, Dco, Dvi, and Pvi groups had significantly higher organic matter contents compared to the KB group, as illustrated in Figure 1b. Figure 1c,f show that the TN and AN contents in the rhizosphere soil of Ceq, Dco, Dvi, and Pvi were significantly greater than those in the rhizosphere of KB, whereas those in Ppl were significantly lower compared to the KB group (*p* < 0.05).

Figure 1d shows that the TP content of the seven plants in this mining area changed significantly, which may be closely related to the planting of different plants. Figure 1g shows that the AP contents of the Dvi and Ceq groups were significantly greater than those of the KB group (*p* < 0.05). Figure 1e,f show the TK content and AK content in the rhizosphere soil of each sample, respectively. Compared with those of the KB group, the TK and AK contents of the rhizosphere soils of the seven plants significantly increased (*p* < 0.05).

#### 3.1.2. Determination of Rhizosphere Soil Metal Element Content

**Figure 2:**
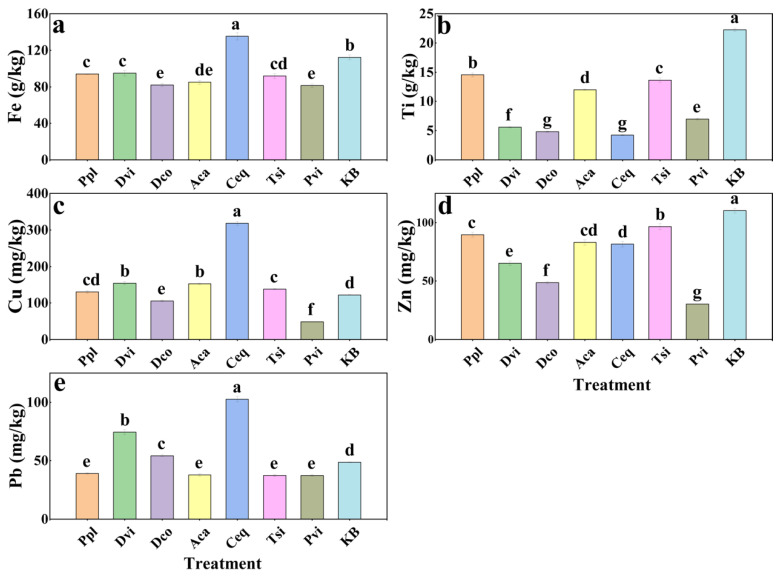
Metal element (Fe (**a**), Ti (**b**), Cu (**c**), Zn (**d**), and Pb (**e**)) contents in rhizosphere soil samples. Different letters indicate significant differences between samples (*p* < 0.05). KB, soil sample from the ilmenite area without plants; Ppl, rhizosphere soil of *Polygonum plebeium*; Dvi, rhizosphere soil of *Dodonaea viscosa*; Dco, rhizosphere soil of *Dryopteris coreano-montana*; Aca, rhizosphere soil of *Alhagi camelorum*; Ceq, rhizosphere soil of *Casuarina equisetifolia*; Tsi, rhizosphere soil of *Tournefortia sibirica*; and Pvi, rhizosphere soil of *Pteris vittate*.

The contents of five metal elements—Fe, Ti, Cu, Zn, and Pb—in the rhizosphere soil were measured to analyze the effects of different plants on the metal element contents in the rhizosphere soil. Figure 2 shows that the Ti and Fe contents of the rhizosphere soils of the seven plants were much greater than the contents of the other metal elements. As can be seen in Figure 2a, compared to the KB group, except for the Ceq group, the Fe content of the rhizosphere soil of the other plants was significantly lower than that of the KB group. Figure 2b shows that all rhizosphere soil samples contained significantly less titanium than the KB group (*p* < 0.05).

It can be seen from Figure 2c–e that the contents of Cu, Zn, and Pb in the rhizosphere soil samples were lower compared to those of the KB group. The Cu contents of Dco and Pvi were lower than those of the KB group, while the Cu contents of the other groups were significantly greater than those of the KB group (*p* < 0.05). However, the Zn content of the rhizosphere soil of the seven plants was significantly lower than that of the KB group. The Ceq group had the highest Pb content in the rhizosphere soil samples, while the Pb contents of the Ppl, Tsi, Aca, and Pvi groups were significantly lower than those of the KB group (*p* < 0.05).

### 3.2. Sequencing Data Analysis

**Figure 3:**
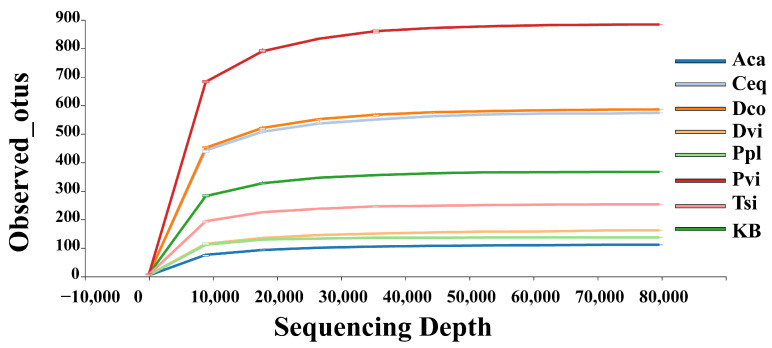
Species rarefaction curves for different rhizosphere soil samples. KB, soil sample from the ilmenite area without plants; Ppl, rhizosphere soil of *Polygonum plebeium*; Dvi, rhizosphere soil of *Dodonaea viscosa*; Dco, rhizosphere soil of *Dryopteris coreano-montana*; Aca, rhizosphere soil of *Alhagi camelorum*; Ceq, rhizosphere soil of *Casuarina equisetifolia*; Tsi, rhizosphere soil of *Tournefortia sibirica*; and Pvi, rhizosphere soil of *Pteris vittate*.

This study analyzed the changes in rhizosphere soil fungal diversity and community structure of seven different plants in the ilmenite area. After completing the quality control steps, an average of 103,792 clean reads were obtained per sample. We plotted the amount of extracted detection data on the horizontal axis and the observed_otus as the vertical axis to create a dilution curve to observe the changes in the sequencing data and observed_otus (Figure 3). The figure demonstrates that as the amount of sequencing data increased, the number of observed_otus in each sample also rose. When the amount of sequencing data exceeded 40,000, the dilution curve gradually stabilized. These results suggest that the sequencing data in this study sufficiently reflect the overall status of the soil fungi in the rhizosphere of each plant. The community structure and diversity are also reflected in the sequencing data.

### 3.3. Fungal Community Analysis

**Figure 4:**
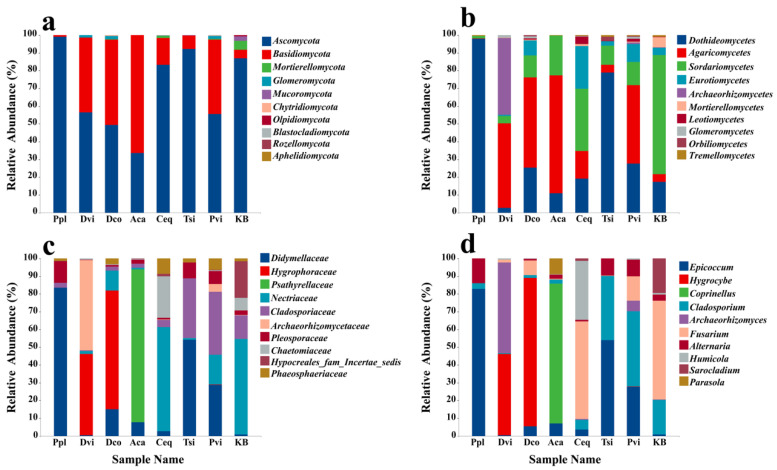
Relative abundance (top 10) of taxa at the phylum (**a**), class (**b**), family (**c**), and genus (**d**) levels in different rhizosphere soil samples. KB, soil sample from the ilmenite area without plants; Ppl, rhizosphere soil of *Polygonum plebeium*; Dvi, rhizosphere soil of *Dodonaea viscosa*; Dco, rhizosphere soil of *Dryopteris coreano-montana*; Aca, rhizosphere soil of *Alhagi camelorum*; Ceq, rhizosphere soil of *Casuarina equisetifolia*; Tsi, rhizosphere soil of *Tournefortia sibirica*; and Pvi, rhizosphere soil of *Pteris vittate*.

A total of 14 fungal phyla were detected across all soil samples. Our study analyzed variations in abundance among the top ten most prevalent phyla (Figure 4a). The figure illustrates that Ascomycota was the most abundant phylum across all soil samples, followed by *Basidiomycota*, *Mortierellomycota*, and *Glomeromycota*. When compared to the KB group, an increase in *Basidiomycota* abundance was observed in the rhizosphere soil of all plants except Ppl, whereas the abundance of *Mortierellomycota* decreased.

A total of 53 fungal classes were identified within all soil samples (Figure 4b), with *Dothideomycetes* being the most abundant, followed by *Sordariomycetes*, *Agaricomycetes*, and *Eurotiomycetes*. Compared to the KB group, the abundance of *Dothideomycetes* in the rhizosphere soil samples significantly increased in all groups except for Aca and Dvi (*p* < 0.05). The number of *Sordariomycetes* in the rhizosphere soil of the seven plants significantly decreased (*p* < 0.05). Compared to those in the control group, the abundance of *Agaricomycetes* in Aca, Ceq, Dco, Pvi, and Dvi also significantly increased (*p* < 0.05). Furthermore, the abundance of *Archaeorhizomycetes* in Dvi exceeded that found in the rhizosphere soil of other plants. This could be due to the planting of *D. viscosa*, which promoted the enrichment of *Archaeorhizomycetes*.

At the family level, a total of 283 fungi were identified in all soil samples (Figure 4c). We compared the abundances of the top ten fungal species, and *Didymellaceae* was the most abundant, followed by *Hygrophoraceae*, *Psathyrellaceae*, and *Nectriaceae*. Compared to those in the control group, the abundances of *Didymellaceae* in Ppl, Tsi, Aca, Dco, and Pvi significantly increased (*p* < 0.05), while the abundances of *Hygrophoraceae* in Dco and Dvi also significantly increased (*p* < 0.05). However, the abundance of *Psathyrellaceae* was significantly greater in Aca than in the other soil samples (*p* < 0.05).

At the genus level, a total of 577 genera and fungi were identified in all of the samples. The rhizosphere soil samples were enriched with *Cladosporium*, *Epicoccum*, *Alternaria*, and *Fusarium*. *Epicoccum* accounted for the highest abundance among all of the samples, followed by *Hygrocybe*, *Corinellus*, and *Cladosporium* (Figure 4d). The figure shows that the fungal genera in the rhizosphere soils of the various plants differed significantly. Compared to those in the control group, the abundances of *Epicoccum* in Ppl and Tsi were greater (*p* < 0.05). The abundance of *Cladosporium* in Tsi was greater (*p* < 0.05), the abundance of *Coprinellus* in Aca was significantly greater (*p* < 0.05), and the abundances of *Hygrocybe* were significantly greater in Dco and Dvi (*p* < 0.05). Compared to those in the KB group, the abundances of *Fusarium* and *Sarocladium* in the rhizosphere soil samples of the seven plants were significantly lower (*p* < 0.05).

### 3.4. Alpha Diversity Index

**Figure 5:**
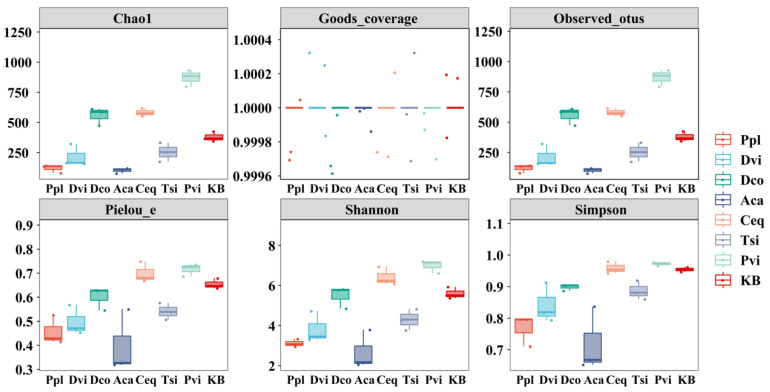
Box plot of alpha diversity index of different rhizosphere soil samples. KB, soil sample from the ilmenite area without plants; Ppl, rhizosphere soil of *Polygonum plebeium*; Dvi, rhizosphere soil of *Dodonaea viscosa*; Dco, rhizosphere soil of *Dryopteris coreano-montana*; Aca, rhizosphere soil of *Alhagi camelorum*; Ceq, rhizosphere soil of *Casuarina equisetifolia*; Tsi, rhizosphere soil of *Tournefortia sibirica*; and Pvi, rhizosphere soil of *Pteris vittate*.

In order to understand the intricacies of species diversity within the rhizosphere soil samples of seven distinct plants, a comprehensive analysis was conducted using six key indicators: Observed_otus, Chao1, Goods_coverage, Shannon index, Simpson index, and Pielou_e (Figure 5). Different plants present different fungal community structures in rhizosphere soils. Compared to those in the KB group, the Observed_otus and Chao1 values of the Ceq, Dco, and Pvi fungi were significantly greater (*p* < 0.05), whereas the Observed_otus and Chao1 values of the Ppl, Tsi, Aca, and Dvi fungi were significantly lower (*p* < 0.05). The Shannon index of Pvi fungi significantly increased (*p* < 0.05), while the Shannon indices of Ppl, Tsi, Aca, and Dvi fungi significantly decreased (*p* < 0.05). Additionally, the Shannon indices of Ceq and Dco increased. Compared to those in the CK group, only the Ppl, Aca, and Dvi fungi showed significantly lower Pielou_e and Simpson indices (*p* < 0.05). The sequencing depth relative to the sequencing depth index (Good’s_coverage) was high for all samples, reaching approximately one.

### 3.5. Microbial Community and Structural Differentiation

**Figure 6:**
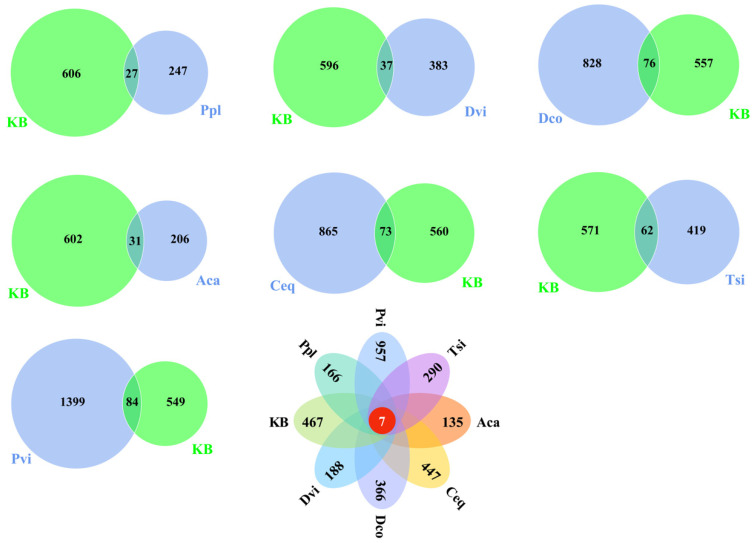
Shared and unique OTUs among different samples. KB, soil sample from the ilmenite area without plants; Ppl, rhizosphere soil of *Polygonum plebeium*; Dvi, rhizosphere soil of *Dodonaea viscosa*; Dco, rhizosphere soil of *Dryopteris coreano-montana*; Aca, rhizosphere soil of *Alhagi camelorum*; Ceq, rhizosphere soil of *Casuarina equisetifolia*; Tsi, rhizosphere soil of *Tournefortia sibirica*; and Pvi, rhizosphere soil of *Pteris vittate*. The numbers in the overlapping areas of the graphs represent ASVs common to the sample soils, and the numbers in the non-overlapping areas represent ASVs specific to the plant sample soils.

This study analyzed the specific ASVs and common ASVs in the rhizosphere soil and soil samples without seven plants (Figure 6). Compared to the KB group, Ppl, Dvi, Dco, Aca, Ceq, Pvi, and Tsi produced 247, 383, 828, 206, 865, 1399, and 499 specific ASVs, respectively. All samples contained seven common ASVs, and each sample contained 135–957 specific ASVs.

**Figure 7:**
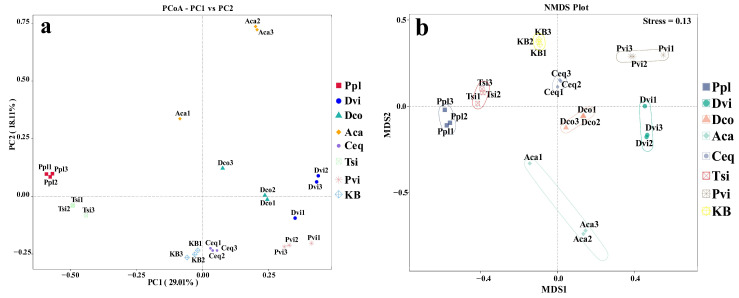
Principal Coordinate Analysis (PCoA) (**a**) and Nonmetric Multidimensional Scaling (NMDS) analysis (**b**) were performed between different samples based on the weighted UniFrac distance. KB, soil sample from the ilmenite area without plants; Ppl, rhizosphere soil of *Polygonum plebeium*; Dvi, rhizosphere soil of *Dodonaea viscosa*; Dco, rhizosphere soil of *Dryopteris coreano-montana*; Aca, rhizosphere soil of *Alhagi camelorum*; Ceq, rhizosphere soil of *Casuarina equisetifolia*; Tsi, rhizosphere soil of *Tournefortia sibirica*; and Pvi, rhizosphere soil of *Pteris vittate*.

We conducted Principal Coordinate Analysis (PCoA) and Nonmetric Multidimensional Scaling (NMDS) on all samples, utilizing the weighted UniFrac distance as the basis for comparison. The community differences in the rhizosphere fungi caused by different plants were investigated. According to PCoA (Figure 7a) and NMDS (Figure 7b), the rhizosphere soil fungal community structures of all seven plant species showed significant differences compared to the KB group.

### 3.6. Prediction of Fungal Community Function

**Figure 8:**
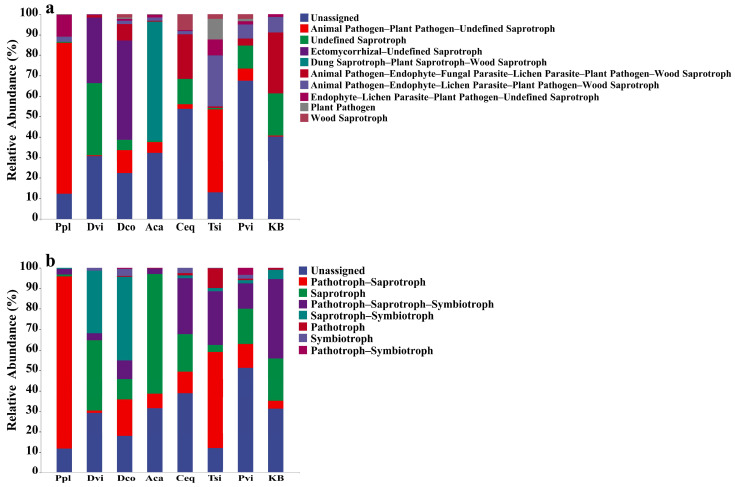
Relative abundance (top ten) of fungal guilds (**a**) and modes (**b**) in different samples based on FunGuild prediction. KB, soil sample from the ilmenite area without plants; Ppl, rhizosphere soil of *Polygonum plebeium*; Dvi, rhizosphere soil of *Dodonaea viscosa*; Dco, rhizosphere soil of *Dryopteris coreano-montana*; Aca, rhizosphere soil of *Alhagi camelorum*; Ceq, rhizosphere soil of *Casuarina equisetifolia*; Tsi, rhizosphere soil of *Tournefortia sibirica*; and Pvi, rhizosphere soil of *Pteris vittate*.

Based on FunGuild’s classification, the soil fungi present in the samples could be categorized into 97 distinct functional groups (Figure 8). Among these guilds, Unassigned accounted for the largest proportion (average 28.01%), and the other guilds included Animal Pathogen–Plant Pathogen–Undefined Saprotroph (average 16.29%), Undefined Saprotroph (average 9.12%), Ectomycorrhizal–Undefined Saprotroph (average 8.71%), and Dung Saprotroph–Plant Saprotroph–Wood Saprotroph (average 7.15%). The abundance of guilds was different in each sample. The trophic modes of all the soil samples were divided into eight types. Among them, the Unassigned trophic mode accounted for the largest proportion (average 28.02%), followed by the other trophic modes, followed by Pathotrophs–Saprotrophs (average 22.76%), Saprotrophs (average 20.27%), Pathotrophs–Saprotrophs–Symbiotrophs (average 15.44%), Saprotrophs–Symbiotrophs (average 10.08%), Pathotrophs (average 1.62%), Symbiotrophs (average 1.16%), and Pathotrophs–Symbiotrophs (average 0.50%).

**Figure 9:**
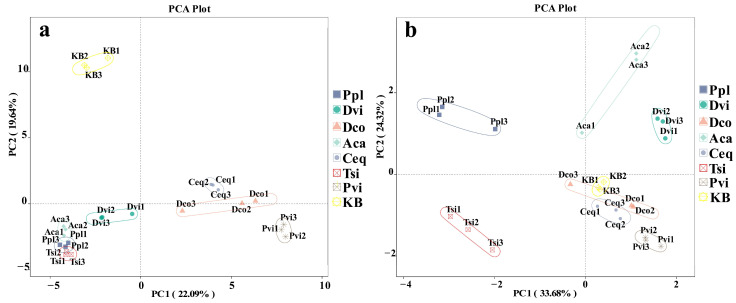
The PCA results of fungal guilds (**a**) and modes (**b**) based on FunGuild prediction. KB, soil sample from the ilmenite area without plants; Ppl, rhizosphere soil of *Polygonum plebeium*; Dvi, rhizosphere soil of *Dodonaea viscosa*; Dco, rhizosphere soil of *Dryopteris coreano-montana*; Aca, rhizosphere soil of *Alhagi camelorum*; Ceq, rhizosphere soil of *Casuarina equisetifolia*; Tsi, rhizosphere soil of *Tournefortia sibirica*; and Pvi, rhizosphere soil of *Pteris vittate*.

We used PCA to analyze changes in fungal guilds and modes in each sample (Figure 9). The results revealed that the guilds of the rhizosphere soil fungi of the seven plant species were more differentiated compared to those of the KB group. In addition, the rhizosphere soil fungi would vary to some extent among the different plants. The modes of Ppl, Aca, Dvi, and Tsi fungi varied to a greater extent compared to the KB group.

### 3.7. Effects of Different Plants in the Mining Area on Guilds and Trophic Patterns

**Figure 10:**
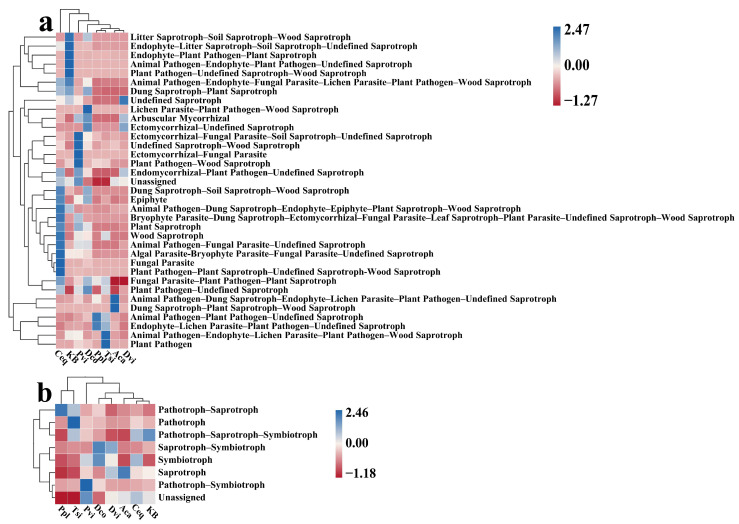
The relative abundance of fungal guilds (**a**) and modes (**b**) predicted by FunGuild in different samples. The relative abundance of different genera is indicated by different colored blocks, with colors ranging from red to blue indicating an increase in the relative abundance. KB, soil sample from the ilmenite area without plants; Ppl, rhizosphere soil of *Polygonum plebeium*; Dvi, rhizosphere soil of *Dodonaea viscosa*; Dco, rhizosphere soil of *Dryopteris coreano-montana*; Aca, rhizosphere soil of *Alhagi camelorum*; Ceq, rhizosphere soil of *Casuarina equisetifolia*; Tsi, rhizosphere soil of *Tournefortia sibirica*; and Pvi, rhizosphere soil of *Pteris vittate*.

The FunGuild prediction results revealed enrichment of Unassigned, Animal Pathogen–Plant Pathogen–Undefined Saprotroph, and Undefined Saprotroph guilds. The guilds enriched by fungi in the rhizosphere soils of different plants differed (Figure 10a). Compared with those of the KB group, the rhizosphere soils of different plants were enriched in different fungal guilds: the Ppl sample enriched in the Animal Pathogen–Plant Pathogen–Undefined Saprotroph and the Endophyte–Lichen Parasite–Plant Pathogen–Undefined Saprotroph guilds; the Tsi sample enriched in the Animal Pathogen–Endophyte–Lichen Parasite–Plant Pathogen–Wood Saprotroph and Plant Pathogen guilds; the Dvi sample enriched in the Undefined Saprotroph guilds; the Aca sample enriched in the Dung Saprotroph–Plant Saprotroph–Wood Saprotroph and Animal Pathogen–Dung Saprotroph–Endophyte–Lichen Parasite–Plant Pathogen–Undefined Saprotroph guilds; the Dco sample enriched in the Lichen Parasite–Plant Pathogen–Wood Saprotroph, Ectomycorrhizal–Undefined Saprotroph, and Plant Pathogen–Undefined Saprotroph guilds; and the Pvi sample enriched in Undefined Saprotrophic–Wood Saprotroph, and Ectomycorrhizal–Fungal Parasite–Soil Saprotroph–Undefined Saprotroph guilds. In addition, different plants also cause different modes of rhizosphere fungi. Compared with those of the KB sample, the fungal mode of the Ppl sample was Pathotroph–Saprotroph; the fungal mode of the Tsi sample was Pathotroph; the fungal mode of the Dvi sample was Saprotroph–Symbiotroph; the main modes of the fungi in the Dco sample were Saprotroph–Symbiotroph and Symbiotroph; the main modes of the Pvi sample were Pathotroph–Symbiotroph and Unassigned; the main mode of the Aca sample was Saprotroph; and the main modes of the Ceq sample were Unassigned and Pathotroph–Saprotroph–Symbiotroph.

Samples of rhizosphere soil fungi were enriched with Unassigned, Pathotroph–Saprotroph, and Saprotroph modes. The modes of rhizosphere soil fungi were significantly altered in seven plants compared to the KB group (Figure 10b). Compared with that in the KB group, the abundance in the Unassigned mode was significantly greater in the Ceq and Pvi groups (*p* < 0.05), whereas the abundance in the Unassigned mode was significantly lower in the Ppl, Dco, and Tsi groups (*p* < 0.05). Compared with that in the KB group, the Pathotroph–Saprotroph mode was significantly lower in the Dvi group (*p* < 0.05), but its abundance was significantly greater in the rhizosphere soil of the other six plants (*p* < 0.05). In addition, compared to the KB group, the abundance of the saprotrophic mode significantly increased in the Dvi and Aca groups (*p* < 0.05) and significantly decreased in the Ppl, Dco, and Tsi groups (*p* < 0.05). In the rhizosphere soil samples of the seven plants, the abundance of the Pthotroph–Sprotroph–Smbiotroph mode of fungi significantly decreased (*p* < 0.05). Compared to the KB group, the abundance of the Sprotroph–Smbiotroph mode significantly increased in the Dvi group and the Dco group (*p* < 0.05), while the abundance of the other groups significantly decreased (*p* < 0.05).

### 3.8. Correlation Analysis of Fungal Community Compositions in Rhizosphere Soils Across Seven Plant Species

**Figure 11:**
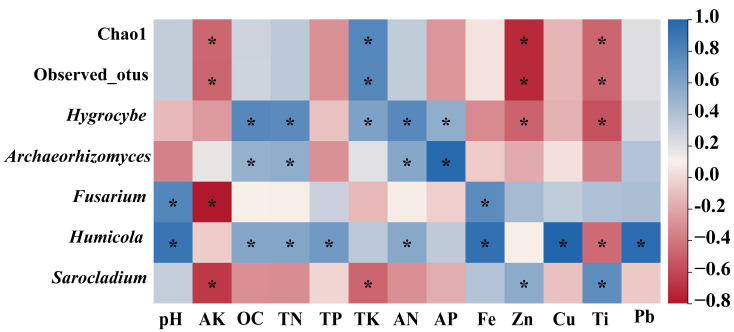
Correlation analysis of alpha diversity index and top ten fungal genera in abundance with soil physicochemical properties. The color bar in the upper right corner represents the correlation coefficient value, with blue representing a positive correlation and red representing a negative correlation. The asterisk indicates that the correlation reached a significant level (*p* < 0.05).

We further analyzed the correlation between the top ten fungal genera in abundance and the alpha diversity index with soil physicochemical properties using Pearson’s correlation coefficient (Figure 11). The figure shows that the abundance of *Hygrocytes* was significantly positively correlated with the contents of OC, TN, TK, AN, and AP, and significantly negatively correlated with the contents of Zn and Ti (*p* < 0.05). In addition, the contents of OC, TN, AN, and AP were significantly positively correlated with the abundance of *Archaeorhizomyces* (*p* < 0.05). The abundance of *Fusarium* was significantly negatively correlated with AK content and significantly positively correlated with pH and Fe content (*p* < 0.05). *Humicola* was significantly negatively correlated with the Ti content and significantly positively correlated with pH, as well as the OC, TN, TP, AN, Fe, Cu, and Pb contents (*p* < 0.05). The presence of *Sarocladium* exhibited a notable positive relationship with Zn and Ti concentrations while displaying a significant negative association with potassium and total potassium levels (*p* < 0.05). It is noteworthy that the alpha diversity coefficient (observed_otus and chao1) showed a significant positive correlation with TK content and a significant negative correlation with AK, Zn, and Ti content (*p* < 0.05).

## 4. Discussion

Plant soil interactions are complex and soils of certain compositions can promote plant growth and, vice versa, plants can improve soil health [47,48]. Our findings indicated variations in the physicochemical characteristics of rhizosphere soils among various plant species [49]. The pH values of the soil samples revealed that the rhizosphere soil of the plants was mostly neutral or slightly alkaline. Compared to those in the KB group, the OC, TN, AN, TK, and AK contents of the rhizosphere soil were greater in the seven planted plants. On the one hand, this may be due to the richer soil nutrients resulting from the degradation of plant litter [50,51]. On the other hand, plant root exudates, such as organic acids, amino acids, and sugars, may provide rich nutrients to the soil [52,53,54]. In addition, plant growth and metabolism require the participation of elements such as iron [55]. Some studies have shown that plants can improve metal-contaminated soil [56]. These observations align with our findings, which show that the metal element contents in the rhizosphere soils of various plants were lower than those in the KB group.

Prior research has indicated that uranium and phosphorus mining affect the distribution and differentiation of fungal community structures [57,58]. Our study revealed that, apart from mining activities in the mining region, the presence of diverse plant species also influences the variation in fungal community composition within the area. We utilized alpha diversity indices to analyze the fungal composition in the rhizosphere soil of seven distinct plant species, as well as in soil without plants. The results showed that *C. equisetifolia*, *P. vittata*, and *D. coreano-montana* increased the abundance of rhizosphere soil fungal communities while diminishing the population of fungal communities in the rhizosphere soil associated with the remaining four plants. This could be attributed to variations in the growth metabolism among different plants as well as differences in the physicochemical characteristics of the soil [59].

Fungal communities play a critical role in plant–soil interactions [60]. In our study, at the phylum level, *Ascomycota* and *Basidiomycota* emerged as the predominant phyla in the soil samples. Many *Ascomycota* species are considered pathogenic and they may contribute to the spread of various soil diseases [60]. *Basidiomycota* species can act as decomposers of plant residues, promoting material cycling in mining areas [61,62]. In addition, the fungal compositions at the genus level were quite different among different plants. The abundance of *Epicoccum* in the rhizosphere soil of *P. plebeium* and *T. sibirica* was significantly greater than that in the other groups. Some studies have shown that *Epicoccum* can not only cause plant diseases but also act as a biological control agent for plant pathogens [63,64]. In addition, the abundance percentage of *Coprinellus* was higher in the rhizosphere soil of *A. camelorum* Fisch. Studies have shown that this fungus can degrade phenols, heavy metals, and other pollutants in the soil [65,66]. This approach is very beneficial for the remediation of soil contamination. On the other hand, in *D. coreano-montana* and *A. camelorum*, a relatively high proportion of *Hygrocytes* were present in the rhizosphere soil. *Hygrocytes* can decompose organic matter in the environment to obtain nutrients, thereby promoting material cycling in the soil [67]. These results all indicate that the rhizosphere soil of different plants enriches some flora that are beneficial to soil remediation. Furthermore, previous studies have revealed that the presence of *Fusarium* in the rhizosphere soil of plants in mining areas increases the risk of various plant diseases [68,69,70]. As a result, compared with those in the KB group, the abundance of *Fusarium* in the rhizosphere soil of the seven plants decreased significantly. This indicates that certain plants can effectively suppress diseases caused by *Fusarium* in this mining area [71,72]. In general, the fungal community of rhizosphere soil is closely related to the plant species. Therefore, in the future, during the microbial remediation of contaminated soils in mining areas, we can choose a specific combination of plants and microbial communities to maximize the effect of soil remediation.

Qiu et al. reported that fungi in different environments belong to different guilds and modes [57]. We used FunGuild to analyze the fungal guilds and modes in the rhizosphere soil of seven plants in this mining area. Those results revealed that different plants could lead to the differentiation of fungal guilds in the rhizosphere soil to a certain extent. Compared with those of the KB sample, the fungal mode of the Ppl sample was Pathotroph–Saprotroph; the fungal mode of the Tsi sample was Pathotroph; the fungal mode of the Dvi sample was Saprotroph–Symbiotroph; the main modes of the fungi in the Dco sample were Saprotroph–Symbiotroph and Symbiotroph; the main modes of the Pvi sample were Pathotroph–Symbiotroph and Unassigned; the main mode of the Aca sample was Saprotroph; and the main modes of the Ceq sample were Unassigned and Pathotroph–Saprotroph–Symbiotroph. These results indicate that different plants can alter the diversity of fungal guilds and modes in the rhizosphere to some extent [73]. Notably, the Animal Pathogen–Plant Pathogen–Undefined Saprotroph guild was enriched in the rhizosphere soil of various plants compared to the KB group. This indicates that different plants can enrich fungi with multiple functions, increasing their ability to remediate soil contaminated by mining areas. In addition, saprotrophic fungi were abundant in the rhizosphere soil samples from this mining area. It suggests that saprotrophic fungi have a crucial role in soil restoration and nutrient recycling within the ilmenite region [74,75,76].

Chu et al. reported that different plants can affect the composition of soil microbes by altering the physicochemical properties of the soil [29]. We correlated the top ten fungal genera in terms of abundance with soil properties. The results revealed that the richness of *Fusarium* and *Humicola* had a significantly positive correlation with soil pH. Additionally, the contents of OC, TN, and AN had a significantly positive correlation with the presence of *Hygocybe*, *Archaeorhizomyces*, and *Humicola*. The results suggest that variations in the fungal communities within the rhizosphere soil of this mining area could be attributed to alterations in soil physicochemical properties resulting from distinct plant species. This is also consistent with previous findings [77]. Studies have also shown that contamination by various metals has a significant effect on microbial communities [78]. Our findings indicated a significant positive correlation between Fe content and the abundance of *Fusarium* and *Humicola* species, while Ti content exhibited a significant positive relationship with the richness of *Sarocladium* and a negative correlation with the abundance of *Humicola* and *Hygrocybe*. These findings suggest that metallic elements are also important drivers of changes in fungal diversity in this mining area [79].

Through the findings above, we have deepened our understanding of the physicochemical properties of rhizosphere soil in different plants and the assembly mechanism of fungal community structure and function in the ilmenite zone. This helps us to screen plant and fungal combinations that are favorable for restoring contaminated soil in the ilmenite zone and provides important technical support for remediating soil pollution in the area.

## 5. Conclusions

This study analyzed the changes in soil properties of the rhizosphere soils of seven different plants planted in the ilmenite area, as well as the assembly mechanisms of the structure and function of the rhizosphere soils of different plants. Using the α diversity index, we found that *C. equisetifolia*, *P. vittata*, and *D. coreano-montana* increased the richness of fungal communities in the rhizosphere soil, while the β diversity index indicated significant differences in fungal community structure in the rhizosphere soil of different plants. It is worth noting that the presence of plants in the mining area resulted in a lower abundance of *Fusarium* in their rhizosphere soil fungi compared to the soil without plants. Additionally, the rhizosphere soil fungi of various plants exhibited varying levels of enrichment. We conducted a correlation analysis on the physicochemical properties and heavy metal contents of the rhizosphere soil and fungal genera. The results revealed that soil physicochemical properties and heavy metal pollution had certain effects on fungal communities. FunGuild’s functional predictions showed that compared to KB group soil fungi, rhizosphere soil fungi of growing plants had Unassigned, Animal Pathogen–Plant Pathogen–Undefined Saprotroph, and Undefined Saprotroph guilds, as well as Unassigned, Pathotroph–Saprotroph, and Saprotroph modes enrichment. These results indicate that plant species, soil physicochemical properties, and heavy metal pollution are important factors affecting fungal communities in the rhizosphere soil. Our study provides a preliminary description of the assembly mechanisms and functional adaptations of fungal communities in the rhizosphere soils of different plants in the ilmenite zone, and the specific mechanisms of action need to be further investigated by considering different plants, different environments, and different fungal characteristics. We still need to improve the theoretical foundation through a large number of experiments, which will provide an important reference for the ecological restoration of ilmenite fields in the future.

## Data Availability

All data analyzed during this study are included in this article.
